# Ant Colony Clustering for ROI Identification in Functional Magnetic Resonance Imaging

**DOI:** 10.1155/2019/5259643

**Published:** 2019-12-26

**Authors:** Alejandro Veloz, Alejandro Weinstein, Stefan Pszczolkowski, Luis Hernández-García, Rodrigo Olivares, Roberto Muñoz, Carla Taramasco

**Affiliations:** ^1^Escuela de Ingeniería Civil Biomédica, Universidad de Valparaíso, Valparaíso 2340000, Chile; ^2^Centro de Investigación y Desarrollo en Ingeniería en Salud, Universidad de Valparaíso, Valparaíso 2340000, Chile; ^3^Radiological Sciences, Division of Clinical Neuroscience, University of Nottingham, Nottingham NG7 2UH, UK; ^4^Functional MRI Laboratory, University of Michigan, Ann Arbor 48109, USA; ^5^Escuela de Ingeniería Civil Informática, Universidad de Valparaíso, Valparaíso 2340000, Chile

## Abstract

Brain network analysis using functional magnetic resonance imaging (fMRI) is a widely used technique. The first step of brain network analysis in fMRI is to detect regions of interest (ROIs). The signals from these ROIs are then used to evaluate neural networks and quantify neuronal dynamics. The two main methods to identify ROIs are based on brain atlas registration and clustering. This work proposes a bioinspired method that combines both paradigms. The method, dubbed HAnt, consists of an anatomical clustering of the signal followed by an ant clustering step. The method is evaluated empirically in both *in silico* and *in vivo* experiments. The results show a significantly better performance of the proposed approach compared to other brain parcellations obtained using purely clustering-based strategies or atlas-based parcellations.

## 1. Introduction

Functional magnetic resonance imaging (fMRI) is a widely used technique in clinical environments for applications such as planning brain surgery or radiation therapy. It is also used in the fields of neuroscience and experimental psychology to discover the complex mechanisms underlying brain function, either through task-related studies or by studying brain networks using resting-state data [1, 2].

The first step of brain network analysis in fMRI data is to detect regions of interest (ROIs). This task is referred to as brain parcellation and aims to divide the brain into a set of nonoverlapping regions that show some homogeneity with respect to the information provided by Blood-Oxygen-Level-Dependent (BOLD) signals. The signals from these ROIs are then used to evaluate neural networks and to quantify neuronal dynamics using network analysis techniques, such as Bayesian networks and Markov models.

A challenge in connectivity analysis is then to determine brain parcellations robustly. Typically, brain parcellation in fMRI is implemented as part of brain network analysis pipelines, with the purpose of organizing or summarizing the high amount of voxel-level information provided by neuroimages into meaningful labels that can be further interpreted and analyzed by researchers. In this regard, there are three strategies commonly used for brain parcellation [3]: (1) the use of seed-based or ROI-based information, (2) the use of a brain atlas, or (3) the use of data-driven parcellations.

The ROI-based or seed-based analysis consists in choosing a set of predefined voxels of interest to conduct brain connectivity analysis [4]. These types of studies often rely on the study of some structure of interest but fail to provide a way to study complex network relationships involving multiple brain nodes.

An alternative approach consists in using brain atlases to define ROIs. This has the advantage that a complete set of ROIs that covers the whole brain volume is provided. There are a number of brain atlases in which brain voxels are organized according to some anatomical, functional, or other predefined criteria. However, using brain atlases often does not fit the data well [3], mainly because atlas parcels are forced to match data, regardless of whether the ROI signals are truly involved in the underlying connectivity network or not. Solving this issue requires a division of the atlas parcels into smaller volumes with more precise functional role.

The third category corresponds to brain parcellations obtained using data-driven strategies, which are commonly derived from clustering algorithms applied to brain images of different modalities [5–7]. The work presented in this paper focuses on this type of method, in particular, on features of BOLD fMRI data [8–11].

Among existing clustering-based parcellation techniques, there are a number of works applying *k*-means variants [8, 12, 13], mixture models [11], hierarchical clustering [14, 15], and spectral clustering [10]. Despite the above approaches have been extensively used in solving the task of finding ROIs in fMRI, more modern ideas from the clustering literature can be incorporated to enhance performance, robustness, and efficiency [16–19].

Although the use of clustering techniques may lead to a grouping of brain voxels in more functionally homogeneous regions based on the measured features of interest [3, 11], one common problem with clustering-based brain parcellations is that they do not directly reflect known anatomical brain structures.

In this work, both the atlas-based and the clustering-based approaches are hierarchically combined with the purpose of finding neuronal groups inside brain atlas labels. For doing this, we take advantage of the bioinspired features of the ant-based colony clustering method [20]. We adapted this ant-based method to find ROIs in fMRI data. We found that this approach improved the performance of the clustering in the task of ROI identification on fMRI compared to other state-of-the-art methods such as the widely used Craddock's approach in connectivity analysis [10].

The assumptions and dynamics followed by ants for grouping data in clustering problems make this approach particularly useful for finding ROIs in fMRI data. First, the ant dynamics works on a two-dimensional grid. We projected the noisy and high-dimensional fMRI signals onto this grid, making the clustering problem easier to solve from a computational point of view. We projected fMRI data such that signals preserve their closeness in terms of their underlying neuronal dynamics and spatial location. The neuronal dynamics closeness was preserved by computing the deconvolution of underlying neuronal events and then projecting those neuronal events into a two-dimensional space. At the same time, the spatial context is preserved by applying this process in labels determined according to a standard brain atlas. Mainly, we used the Talairach labels to perform the aforementioned process [21]. Another feature of the proposed method is that the number of ROIs is determined automatically using a density-based criterion computed on a *k*-neighbours graph structure, which is determined when the fMRI is projected onto the ant's two-dimensional grid.

Ant-inspired algorithms have been successfully applied in time series clustering problems [22, 23]. Similar approaches have been applied in fMRI analysis to select the most informative voxels, which are then used to identify response patterns based on the connectivity of them [24]. Thus, our motivation for using an ant-based clustering algorithm for finding ROIs in fMRI data rests in two main points. First, ants act as agents that group neighboring voxels that share similar temporal dynamics. This allows us to build groups of voxels that maintain the anatomical consistency of ROIs that is needed to apply further brain connectivity analyses. Second, this anatomical consistency is enhanced by the constraint imposed to the ants to group voxels belonging to an anatomical label given by the standard anatomical parcellations.

The contributions of our work are

- A new approach, based on the ant clustering computational paradigm, for discovering ROIs that makes less assumptions about anatomical restrictions of functional areas involved in the brain neuronal dynamics.
- A method that takes advantage of standard brain labels to group voxels, allowing more meaningful and interpretable results. This is relevant to clinicians and neuroscience researchers, who need to interpret results using the same terminology used in standard brain parcellations.
- A method that considers intravoxel temporal dynamics, allowing the exclusion of voxels not belonging to the connectivity network that share the same label.

This paper is structured as follows: Section 2 explains the details regarding the proposed method with the features described above.  Sections 3 and 4 present the results obtained with the proposed method on simulated Dynamic Causal Model data and on *in vivo* fMRI data, respectively. Finally, Section 5 presents some discussion and conclusion about the results obtained and future work.

## 2. Hierarchical Ant Colony Clustering Algorithm for Functional MRI

**Figure 1:**
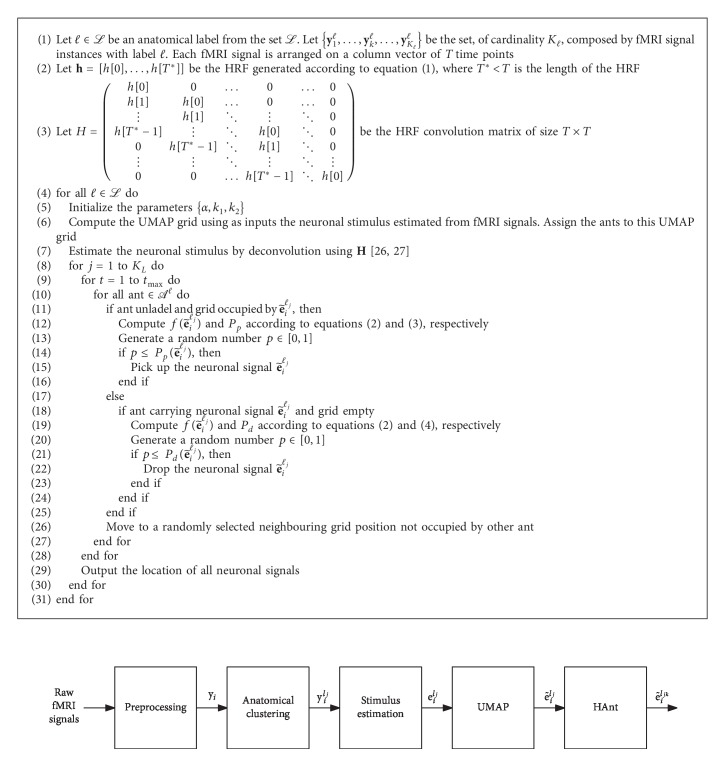
HAnt colony clustering pipeline for fMRI.

In this work, we propose a hierarchical ant-based clustering algorithm, called HAnt, which can be used to determine regions of interest on fMRI. The algorithm consists of five steps, shown in Figure 1. Starting from the raw BOLD fMRI signals, the first step of the algorithm is to apply the standard preprocessing techniques to the signals [25]. The output of this step is the set of signals *𝒴*={**y**_1_,…, **y**_*N*_}, with *N* the number of voxels, **y**_*i*_ ∈ *ℝ*^*T*^, and *T* the number of samples in each fMRI signal. Next, each fMRI signal goes trough an anatomical clustering step. Here, a label is attached to each signal according to the location of the corresponding voxel in a given anatomical atlas. Our implementation uses the Tailarach atlas [21], but any other brain anatomical parcellation can be used. The output of this step is fMRI signals **y**_*i*_^*ℓ*_*j*_^, where *ℓ*_*j*_ ∈ *ℒ*={*ℓ*_1_,…, *ℓ*_*L*_} denotes the label attached to the signal and *L* is the number of labels in the atlas. These labels are later used to constrain the HAnt clustering to regions with known brain histological regularity. The algorithm continues with a stimulus estimation step. This step applies a deconvolution technique to estimate the underlying events or stimulus that generated the observed BOLD signals [26–28]. This is useful because estimated events have a much better signal to noise ratio and also because brain activity is ultimately underpinned by these neural events rather than the indirect observation of the BOLD signal. The process is based on the assumption that the BOLD response is the output of a linear time-invariant (LTI) system [29–32]. We follow the standard assumption that the impulse response of this system is the canonical Glover's haemodynamic response function (HRF) [33], given by

(1) $$\begin{array}{c} h\left(t\right)={\left(\frac{t}{\tau_{1}}\right)}^{\delta_{1}}\exp\left[-\frac{\delta_{1}}{\tau_{1}}\left(t-\tau_{1}\right)\right]-c{\left(\frac{t}{\tau_{2}}\right)}^{\delta_{2}}\exp\left[-\frac{\delta_{2}}{\tau_{2}}\left(t-\tau_{2}\right)\right], \end{array}$$

with parameters {*τ*_1_=5.4, *τ*_2_=10.8, *δ*_1_=6, *δ*_2_=12, *c*=0.35} [29, 34]. The output of this step is the neural event signals **e**_*i*_^*ℓ*_*j*_^ ∈ *ℝ*^*M*^ of voxel *i* with anatomical label *ℓ*_*j*_. To manage the high dimensionality of the BOLD and corresponding neural event signals, the next step projects the data into a two-dimensional space using Uniform Manifold Approximation and Projection (UMAP) [35]. This technique is particularly appropriate to the problem at hand since it excels at preserving the global structure of the data. The output of this step is the projection of the neural event signals ${\tilde{e}}_{i}^{\ell_{j}}\in {\mathbb{R}}^{2}$. The last step of the algorithm consists in applying the ant clustering algorithm to the 2-dimensional neural event signals. A detailed description of this step follows.

We propose an ant-based clustering approach based on the work of Zhang and Cao [20], which is an extension of the algorithm proposed by Lumer and Faieta [36]. The approach is predicated on the way ants cluster corpses to form cemeteries [20]. Under this paradigm, for each anatomical region, a group of ants clusters the signals belonging to that region. Each ant chooses a signal at random and picks up, moves, and drops the signal according to a picking-up or dropping probability. This probability is computed according to a similarity criterion of the current signal with respect to the neighbouring signals. For an ant located at site *r* of the two-dimensional space at time *t*, the local similarity measure of ${\tilde{e}}_{i}^{\ell_{j}}$ is given by [20]

(2) $$\begin{array}{c} f\left({\tilde{e}}_{i}^{\ell_{j}}\right)=\text{max}\left\{0,\frac{1}{s^{2}}\underset{{\tilde{e}}_{k}^{\ell_{j}}\in N_{s\times s}\left(r\right)}{\sum }\left[1-\frac{d\left({\tilde{e}}_{i}^{\ell_{j}},{\tilde{e}}_{k}^{\ell_{j}}\right)}{\alpha \left(1+\left(v-1/v_{\text{max}}\right)\right)}\right]\right\}, \end{array}$$

where parameter *α* is used to adjust the scale of the similarity between objects and parameters *v* and *v*_max_ define the speed and the maximum speed of the ants, respectively. The speed *v* of the ant is chosen randomly from a uniform distribution of range (1, *v*_max_). The term **N**_*s*×*s*_(*r*) denotes a square neighbourhood of size *s* × *s* centered at site *r*, and $d\left({\tilde{e}}_{i}^{\ell_{j}},{\tilde{e}}_{k}^{\ell_{j}}\right)$ is the distance between two objects ${\tilde{e}}_{i}^{\ell_{j}}$ and ${\tilde{e}}_{k}^{\ell_{j}}$ in the space of attributes, which typically correspond to the Euclidean distance.

The probability of an ant picking up a signal is

(3) $$\begin{array}{c} P_{p}\left({\tilde{e}}_{i}^{\ell_{j}}\right)={\left(\frac{k_{1}}{k_{1}+f\left({\tilde{e}}_{i}^{\ell_{j}}\right)}\right)}^{2}, \end{array}$$

where *k*_1_ is a constant. Similarly, the probability of an ant dropping a signal is

(4) $$\begin{array}{c} P_{d}\left({\tilde{e}}_{i}^{\ell_{j}}\right)=\left\{\begin{array}{cc} 2f\left({\tilde{e}}_{i}^{\ell_{j}}\right), & \text{ when }f\left({\tilde{e}}_{i}^{\ell_{j}}\right)<k_{2},\\ 1, & \text{ when }f\left({\tilde{e}}_{i}^{\ell_{j}}\right)\geq k_{2}, \end{array}\right. \end{array}$$

where *k*_2_ is a constant. Our HAnt method is described in full in Algorithm 1, and its time complexity (this time complexity does not include the time complexity of the UMAP and deconvolution preprocessing steps) is *𝒪*(*t*_max_|*𝒜*^*ℓ*^|*T*^2^), which is in line with the time complexity of ant clustering-based algorithms [37].

## 3. Experimental Evaluation with Simulated Data

The quality of the obtained ROIs was investigated on BOLD fMRI synthetic data by computing the silhouette and Davies–Bouldin scores. These scores allow us to determine the consistency of a partition made with a clustering algorithm. In a nutshell, the silhouette score is a measure of how similar a data sample is to the cluster that it was assigned by the clustering algorithm compared to other clusters. The silhouette score ranges within the [−1,1] interval, where a value towards 1 indicate that the fMRI signals are well matched to their own clusters and are poorly matched to neighbouring clusters. Similarly, the Davies–Bouldin score indicates the similarity of clusters, which are assumed to have a data density, which is a decreasing function of distance from a vector characteristic of the cluster. The lower the Davies–Bouldin score is, the better the obtained partition is.

We compare our proposed method (labeled as HAnt) and the widely used Craddock's approach [10]. As mentioned earlier, this approach is based on spectral clustering and has been implemented within many software packages used to find ROIs (see, for example, https://nilearn.github.io/index.html).

The simulated BOLD fMRI data were generated using a Dynamic Causal Model (DCM). A DCM is a model of neural dynamics in a network of *m* brain regions [38]. The neuronal dynamics are generated according to the expression:

(5) $$\begin{array}{c} \dot{x}=Ax+\overset{m}{\underset{j=1}{\sum }}x_{j}B^{\left(j\right)}x+Cu, \end{array}$$

where **x**′ is a column vector of node's time courses and $\dot{x}$ are their temporal derivatives. The diagonal entries of **A** (which are set to −1) determine the node's temporal decay, and the off-diagonal entries are positive numbers that represent the connections between nodes. The matrices **B**^(*j*)^ correspond to nonlinear relations between time courses of different nodes of a network. These matrices encode the modulation (coupling) between nodes. In our experiments, we considered a five-node network with **x**=(*x*_1_, *x*_2_, *x*_3_, *x*_4_, *x*_5_)′.

Similarly, the term **u**=(*u*_1_, *u*_2_, *u*_3_, *u*_4_, *u*_5_)′ is a vector of independent random binary sequences that represent external inputs (stimuli) for the network and **C** determines how these external inputs feed into the network. We chose matrix **C** as the identity, and we set the onsets and duration for the stimulus epoch in such a way that they allow the BOLD signal to return to its baseline before the next stimulus onset.

**Figure 2:**
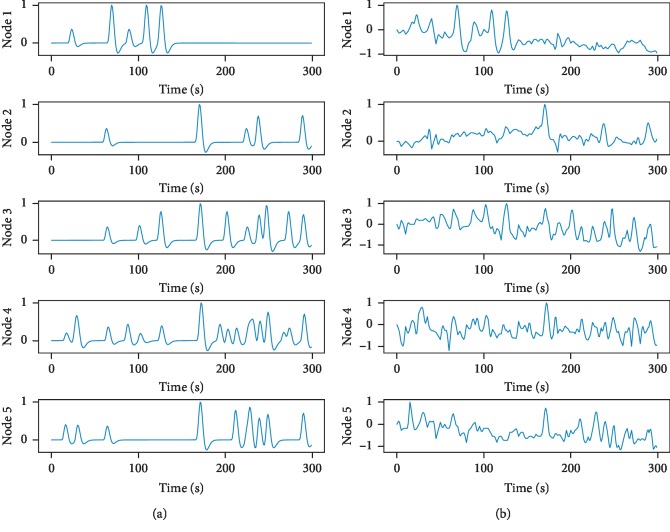
DCM signals before (a) and after (b) noise addition. The SNR of this example was 0.8.

An example of time courses of a five-node DCM for an SNR of 0.8 is shown in Figure 2. In our experiments, the noiseless DCM time courses were replicated 1000 times and then random noise was added to each signal instance. To contaminate the BOLD signals with realistic noise realizations, we used real fMRI data from subjects one, two, and three of the “human voice areas dataset.” More details regarding this dataset are provided in Section 4.

**Figure 3:**
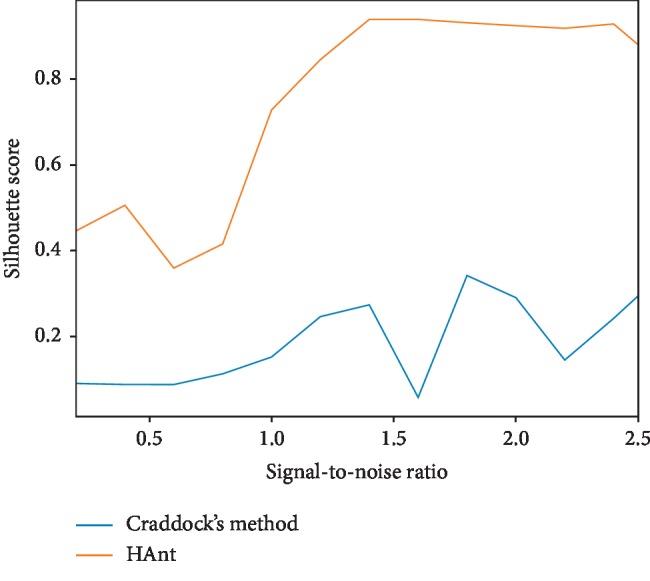
Silhouette scores obtained for different SNRs.

**Figure 4:**
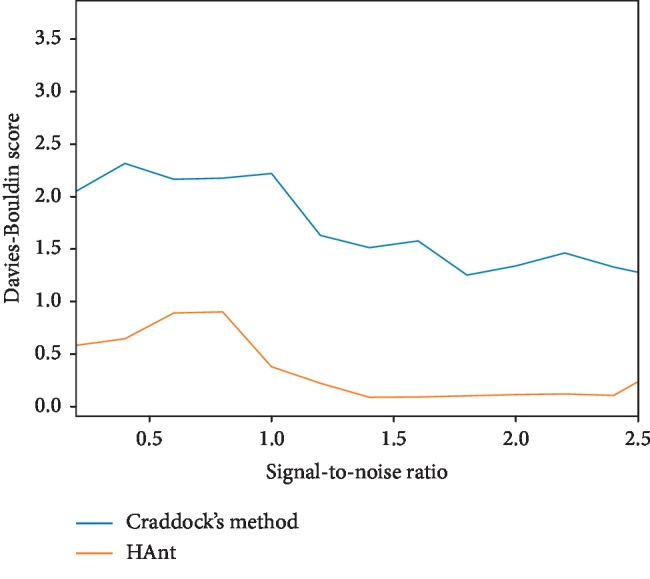
Davies–Bouldin scores obtained for different SNRs.

To add noise, we performed a standard GLM analysis on the data and selected “non-active” brain voxels where the null hypotheses were accepted. We randomly selected noise realizations from this set. To control the signal to noise ratio (SNR), defined as SNR=(*σ*_signal_/*σ*_noise_), we first standardized the DCM and noise signals. Then, these two signals were combined by multiplying the noiseless DCM signal by the desired SNR, to then add the noise realization. We studied the effect of different SNRs (see Figures 3 and 4) (the code of this experiment is available at https://gitlab.com/HAnt_paper_code/).

For our experiments, we set the parameters {*α*, *k*_1_, *k*_2_} to {1.5, 1.1, 1.0}, respectively. The values for this set of parameters were found using a grid search strategy over the parameter product space [0.1, 2] × [0.1, 2] × [0.1, 2] with a step size of 0.1.

Figures 3 and 4 show both the silhouette and Davies–Bouldin scores, respectively, as a function of the SNR.

## 4. Experimental Evaluation with In Vivo fMRI Data

This section describes the results obtained by applying the HAnt method on *in vivo* fMRI data from the human voice areas dataset [25]. Studies conducted using this data aimed to demonstrate the existence of areas in the human auditory cortex sensitive to sounds of voices versus other categories of nonvocal sounds such as scrambled voices and amplitude-modulated noise. It must be noted that this is a research example in which researchers lack *a priori* knowledge about which ROIs would be involved in the perception of sounds. This dataset is freely available in the OpenfMRI database (https://openfmri.org) under the accession number ds000158. Data correspond to 218 healthy adult volunteers (117 males; age 24.1 ± 7.0 (mean ± Std. Dev.)).

Data preparation was performed according to the usual pipeline used in fMRI, i.e., motion and slice-timing correction, spatial smoothing with an edge preserving Gaussian filter, and temporal smoothing with a Gaussian filter. Time courses were also detrended using a third-order polynomial and normalized to unity. The images were then spatially normalized to the Montreal Neurological Institute's MNI152 template using SPM12 (http://www.fil.ion.ucl.ac.uk/spm/).

Additionally, the labels corresponding to each voxel in the normalized brains were found using the Talairach Daemon tool (http://www.talairach.org/daemon.html), in order to obtain the anatomical labels that HAnt needs to initialize the clustering process.

The results obtained by applying HAnt expressed in terms of the silhouette and Davies–Bouldin scores were compared with the corresponding silhouette and Davies–Bouldin scores computed according to Craddock's brain parcellations [10] from the Athena Pipeline of the ADHD 200 preprocessing initiative. These brain parcellations correspond to two atlases of 200 and 400 ROIs, publicly available in https://ccraddock.github.io/cluster_roi/atlases.html.

Finally, the average time of execution is reported to provide insights regarding the computational burden of the proposed method compared to Craddock's method (as shown in Table 1).

It must be noted that the number of ROIs for Craddock's templates and Talairach templates remains the same.

## 5. Discussion and Conclusion

The aim of this work is to develop a new framework for discovering ROIs for the analysis of networks on fMRI data. This new approach makes less assumptions about anatomical restrictions of functional areas involved in the brain neuronal dynamics. We evaluated the performance of the proposed method with both simulated and *in vivo* data. We found that the performance of the proposed approach is substantially better than other brain parcellations obtained using purely clustering-based strategies or atlas-based parcellations.

As observed in the first experiment with synthetic data, our method behaves as expected. The performance of the proposed HAnt clustering tends to optimal values as the noise dissipates from the data, both in terms of silhouette and Davies–Bouldin scores (see Figures 3 and 4). It must be noticed, however, that the performance of Craddock's approach did not improve in the same proportion. This can be attributed to numerical instabilities that are inherent to methods that resort to matrix decomposition techniques, such as spectral clustering approach, when used with high-dimensional datasets.

**Figure 5:**
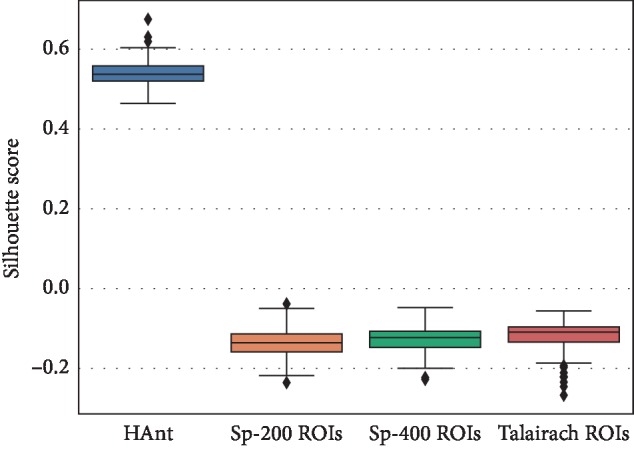
Silhouette scores obtained for the compared methods: HAnt, spectral clustering with 200 ROIs (Sp-200 ROIs), spectral clustering with 400 ROIs (Sp-400 ROIs) and with the Talairach parcellation.

**Figure 6:**
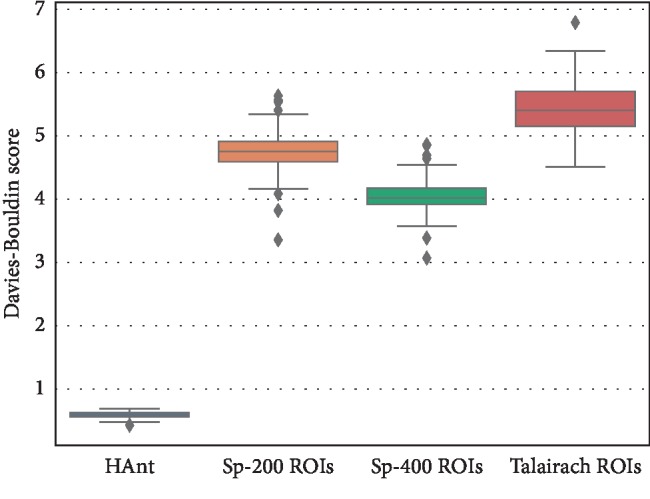
Davies–Bouldin scores obtained for the compared methods: HAnt, spectral clustering with 200 ROIs (Sp-200 ROIs), spectral clustering with 400 ROIs (Sp-400 ROIs), and with the Talairach parcellation.

**Figure 7:**
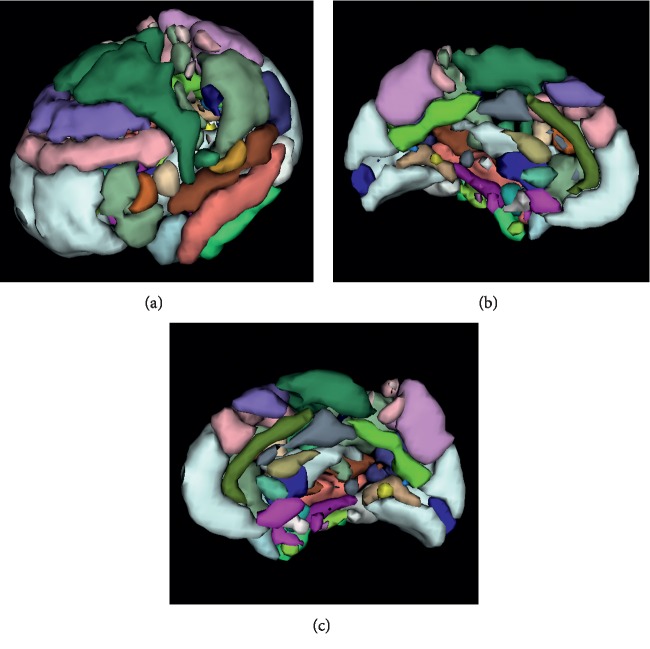
ROIs obtained for the subject one of the human voice areas dataset using the proposed HAnt algorithm.

In the experiments performed on *in vivo* data, the performance of HAnt in terms of both the silhouette and Davies–Bouldin scores is substantially better than Craddock's method (see Figures 5 and 6). In this case, we used the brain parcellations of 200 and 400 ROIs, as provided by the authors. We additionally used the Talairach parcellation to fix the baseline. We can observe from the silhouette and Davies–Bouldin scores that Craddock's methods approach the Talairach parcellation but use a considerably higher number of ROIs. The number of ROIs obtained with HAnt approaches that of Craddock's templates of 200 ROIs but have much more homogeneous ROIs (Figure 7).

Future work will focus on a better parameter setting approach. This may be done by using evolutionary strategies or by using the ensemble clustering methods. These strategies will contribute to further improving the results presented in this paper. Under this approach, instead of using a single HAnt algorithm to produce a single clustering result, a set of ROIs are produced by different HAnt solutions (with different parameters), which are combined into a more robust clustering consensus [19].

## Figures and Tables

**Algorithm 1:**
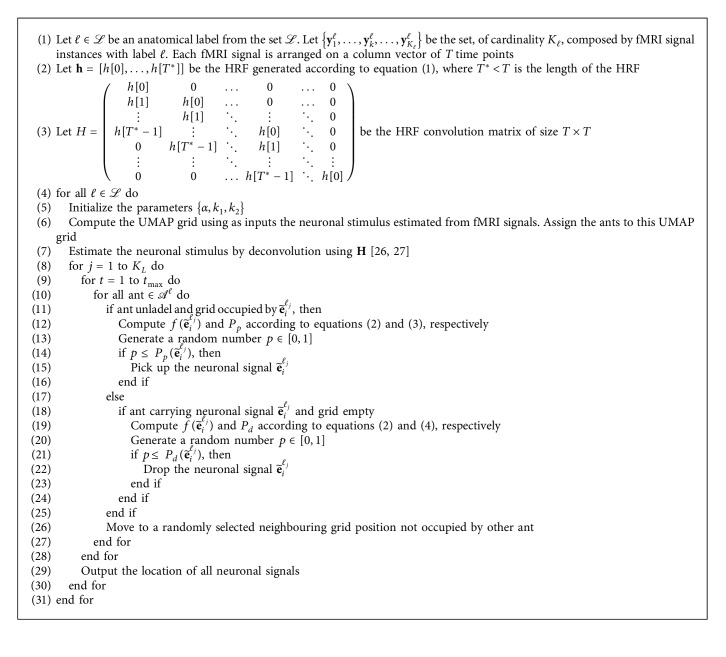
HAnt algorithm for ROI identification in fMRI.

**Table 1:** Average silhouette score, Davies–Bouldin score, time of execution (in seconds), and number of ROIs (± standard deviation) obtained for the human voice areas dataset.

Method	Silhouette score	Davies–Bouldin score	Time (s)	N ROIs
HAnt	0.54 ± 0.04	0.59 ± 0.05	1265.25 ± 58.66	168.81 ± 8.84
Sp-200 ROIs	−0.14 ± 0.04	4.76 ± 0.28	—	190
Sp-400 ROIs	−0.13 ± 0.03	4.06 ± 0.23	—	351
Talairach	−0.12 ± 0.03	5.43 ± 0.38	—	58

## Data Availability

The human voice areas data used to support the findings of this study are freely available in the OpenfMRI database (https://openfmri.org) under the accession number ds000158. Data correspond to 218 healthy adult volunteers (117 males; age 24.1 ± 7.0 (mean ± Std. Dev.)).
